# Vagal-α7nAChR signaling promotes lung stem cells regeneration via fibroblast growth factor 10 during lung injury repair

**DOI:** 10.1186/s13287-020-01757-w

**Published:** 2020-06-10

**Authors:** Xiaoyan Chen, Caiqi Zhao, Cuiping Zhang, Qingmei Li, Jie Chen, Lianping Cheng, Jian Zhou, Xiao Su, Yuanlin Song

**Affiliations:** 1 Department of Pulmonary and Critical Care Medicine, Zhongshan Hospital, Fudan University and Shanghai Respiratory Research Institute, 180 Fenglin Road, Shanghai, 200032 People’s Republic of China; 2 Unit of Respiratory Infection and Immunity, Institut Pasteur of Shanghai, Chinese Academy of Sciences, 320 Yueyang Road, Shanghai, 200031 People’s Republic of China; 3 Department of Pulmonary Medicine, Zhongshan Hospital, Qingpu Branch, Fudan University, Shanghai, People’s Republic of China; 4 National Clinical Research Center for Aging and Medicine, Huashan Hospital, Fudan University, Shanghai, People’s Republic of China

**Keywords:** Vagus nerve, Lung stem cells (LSCs), Multipotential differentiation, Proliferation

## Abstract

**Background:**

Proliferation and transdifferentiation of lung stem cells (LSCs) could promote lung injury repair. The distal airways of the lung are innervated by the vagus nerve. Vagal-alpha7 nicotinic acetylcholine receptor (α7nAChR) signaling plays a key role in regulating lung infection and inflammation; however, whether this pathway could regulate LSCs remains unknown.

**Methods:**

LSCs (Sca1^+^CD45^−^CD31^−^ cells) were isolated and characterized according to a previously published protocol. α7nAChR knockout mice and wild-type littermates were intratracheally challenged with lipopolysaccharide (LPS) to induce lung injury. A cervical vagotomy was performed to study the regulatory effect of the vagus nerve on LSCs-mediated lung repair. α7nAChR agonist or fibroblast growth factor 10 (FGF10) was intratracheally delivered to mice. A single-cell suspension of lung cells was analyzed by flow cytometry. Lung tissues were collected for histology, quantitative real-time polymerase chain reaction (RT-PCR), and immunohistochemistry.

**Results:**

We found that LSCs maintained multilineage differentiation ability and transdifferentiated into alveolar epithelial type II cells (AEC2) following FGF10 stimulation in vitro. Vagotomy or α7nAChR deficiency reduced lung Ki67^+^ LSCs expansion and hampered the resolution of LPS-induced lung injury. Vagotomy or α7nAChR deficiency decreased lung FGF10 expression and the number of AEC2. The α7nAChR agonist-GTS-21 reversed the reduction of FGF10 expression in the lungs, as well as the number of Ki67^+^ cells, LSCs, Ki67^+^ LSCs, and AEC2 in LPS-challenged vagotomized mice. Supplementation with FGF10 counteracted the loss of Ki67^+^ LSCs and AEC2 in LPS-challenged α7nAChR knockout mice.

**Conclusions:**

The vagus nerve deploys α7nAChR to enhance LSCs proliferation and transdifferentiation and promote lung repair in an FGF10-dependent manner during LPS-induced lung injury.

## Background

Evidence indicates that lung resident stem cells possess a proliferative capacity in vivo, and they are activated and subsequently proliferate and differentiate into lung epithelium to effectively regenerate and repair an injured lung [1–3]. Failure to regenerate the lung epithelium contributes to poor resolution in lung diseases such as pneumonia and acute respiratory distress syndrome (ARDS), and it leads to pulmonary scarring and fibrosis [4]. Thus, the reparative capacity of lung stem cells has attracted considerable research attention in terms of both elucidating the mechanism underlying regeneration after lung injury and developing therapeutic strategies for treating injured lungs.

In the adult mouse lung, the stem cell marker (Sca1) has previously been identified as a vital marker for the isolation of candidate nonendothelial (CD31^−^), nonhematopoietic (CD45^−^) bronchioalveolar stem cells (BASCs) located at the bronchioalveolar duct junction that can self-renew over multiple passages and differentiate into airway and alveolar epithelium [1]. However, McQualter et al. reported that lung fibroblastic progenitor cells are highly enriched in the Sca1^+^CD45^−^CD31^−^ population of cells [5]. Hegab et al. revealed that lung resident Sca1^+^CD45^−^CD31^−^ cells exhibited extensive self-renewal capacity and can differentiate into lung epithelial cells (alveolar epithelial type I cells: AEC1, type II cells: AEC2, and club cells) and endothelial cells, and they even exhibited some mesenchymal ability [6]. Although there is divergence regarding their differentiation capacity, lung resident Sca1^+^ CD45^−^CD31^−^ cells have been consistently found to have stem cell properties and contribute to lung regeneration [1, 5–7].

During regeneration processes, the lung stem cell niche plays a key role in initiating and coordinating self-renewal and terminal differentiation. Signals involved in the cross-talk between LSCs and the microenvironmental niche include paracrine fibroblast growth factors (FGFs), which regulate cell proliferation, differentiation, motility, and survival. In particular, FGF10, which is expressed in the adjacent distal lung mesenchyme, binds to and activates fibroblast growth factor receptor 2 (FGFR2) on epithelial progenitors to direct distal lung development, including branching morphogenesis and regeneration after damage [8–12]. FGF10 signaling could be reactivated in the adult lung after injury to regenerate the epithelium [2, 11]. The exact signal triggering the FGF10 network in the context of lung repair after LPS induced injury, however, is not well understood.

The vagal nerve, the main dominant nerve of the distal airway of the lung, which contains alveoli [13, 14], is known to be regulators of both respiratory clocks [15] and local lung immunity [16]. Our group [13, 17, 18] and other groups [19–23] have reported that vagotomy or deletion of alpha 7 nicotinic acetylcholine receptor (α7nAChR), the receptor for a vagal neurotransmitter, significantly amplifies inflammatory responses and exacerbates tissue damage that occurs during infection, endotoxemia, and radiation-induced lung injury. In addition, several studies have suggested that vagal signals are involved in the proliferation, differentiation, and function of various stem cells [24–27]. However, whether they regulate lung resident stem cells during the reparative phase of lung injury has not been clarified. In this study, we investigated whether and how vagal-α7nAChR signaling regulates LSCs to participate in the reparative process of lung injury.

## Methods

### Chemicals

Lipopolysaccharides (LPS) were obtained from *Pseudomonas aeruginosa* (L9143). Dispase II, collagenase IA, deoxyribonuclease I, penicillin/streptomycin/amphotericin B, and insulin/transferrin/selenium were purchased from Sigma-Aldrich (St Louis, MO, USA). GTS-21 dihydrochloride (DMBX-A) (ab120560), a specific α7nAChR agonist, was purchased from Abcam (Cambridge, MA, USA). FGF10 was provided by Newsummit, Shanghai, China. Anti-mouse CD16/CD32 and phycoerythrin (PE) rat anti-mouse/human CD44 monoclonal antibodies (IM7) were purchased from eBioscience (San Diego, CA, USA). Fixable viability stain 780 (FVS780), allophycocyanin (APC) rat anti-mouse CD45 (clone 30-F11), APC rat anti-mouse CD31 (clone MEC 13.3), and PE rat anti-mouse Ly-6A/E (clone D7) were obtained from BD Biosciences (San Jose, CA, USA). PE/Cyanine 7 Armenian hamster anti-mouse/rat (clone HMβ1-1) CD29, PE rat anti-mouse CD105 (clone MJ7/18), and fluorescein isothiocyanate (FITC) anti-mouse TER-119/erythroid cells (clone TER-119) were obtained from Biolegend (San Diego, CA, USA). A rabbit anti-FGFR2 antibody was purchased from Abcam (Cambridge, MA, USA).

### Animals

α7nAChR knockout mice (α7nAChR^−/−^, background, C57BL/6J, B6.129S7-*Chrna7*tm1Bay/J, stock no. 003232) were purchased from Jackson Laboratory (Bar Harbor, ME, USA) [13]. Littermate wild-type mice (α7nAChR^+/+^, background, C57BL/6J, 6–8 weeks old) were used as controls. The mice were housed with free access to food and water in 12-h dark/light cycles. Anesthetization was induced by intraperitoneal injection of pentobarbital sodium (80 mg/kg). Subsequently, FGF10 (5 mg/kg) dissolved in phosphate-buffered saline (PBS) or PBS with equal volume was intratracheally delivered. Three days later, mice were intratracheally injected with 2.5 mg/kg of LPS dissolved in PBS. Mice were humanely sacrificed by lethal overdose of pentobarbital sodium on the 7th day. Animal experiments were approved by the Committees on Animal Research of the Institut Pasteur of Shanghai, Chinese Academy of Sciences, China. The protocols were performed in accordance with the United States National Institutes of Health Guide for the Care and Use of Laboratory Animals (8th Edition, 2011).

### Unilateral vagotomy

Cervical vagotomies were performed as described previously [28]. Briefly, a longitudinal midline incision was made in the ventral region of the neck before blunt dissection. The overlying muscles and fascia were separated until the right vagus was visible. For the vagotomy (Vx) group, the vagus was carefully stripped away from the carotid artery and was carefully cut. For the sham group, the vagus was kept intact. The wound was closed and sutured.

### Animal treatments

After undergoing vagotomy, mice were intratracheally injected with either the indicated concentration (2.5 or 5 mg/kg) of LPS dissolved in PBS or an equal volume of PBS. Subsequently, GTS-21 (4 mg/kg), FGF10 (5 mg/kg), or PBS was intratracheally delivered.

### Histological analysis

Mouse lung sections were stained with hematoxylin-eosin to determine the extent of injury. The lung injury was graded in a blinded manner as described previously [29]: 0 = no injury; 1 = area of lung injury less than 25%; 2 = area of lung injury between 25 and 50%; 3 = area of lung injury between 50 and 75%; and 4 = area of lung injury more than 75%. Immunohistochemistry was performed with an anti-prosurfactant protein C (proSP-C) antibody (MeckMillipore, Darmstadt, Germany) or an anti-FGF10 antibody (ABclonal, Boston, USA). Sections were covered with a DAB tetrahydroxychloride solution and then were counterstained with hematoxylin. The numbers of SP-C-positive cells were counted in 6 random fields (× 200), and the results were expressed as the average number of SP-C-positive cells/lung nucleated cells from 3 or more animals in each group. Scoring of FGF10 protein expression was determined by the hybrid scoring system (H-score) [30]. The intensity of the staining was classified into 4 grades: 0, no staining; 1, weak staining; 2, moderate staining; and 3, strong staining, and then score was multiplied by the percent of positive cells in the area. All the scores were performed in a blinded manner.

### Flow cytometry

After preincubating for 15 min with anti-mouse CD16/32 antibodies, lung cells were labeled with primary or isotype control antibodies. Isotype antibodies and unstained controls were set to demonstrate specificity of staining and to establish the criteria for target populations (for simplicity, data were not shown regarding these controls). Debris and aggregates were excluded, and live cells were analyzed by LSRFortessa (BD Biosciences, San Jose, CA, USA). Data were analyzed by FlowJo vX.0.7 software (Tree Star Inc., Ashland, OR, USA).

### Isolation of lung stem cells

Single-cell suspensions of lung tissue were prepared for each experiment using 3–5 mice as described [5] with modification. In brief, 1 ml of dispase (2 U/ml) was injected through the trachea. Subsequently, the trachea was removed, and the lungs were minced and incubated in a 37 °C shaking incubator for 45 min in 2 ml of 2 μg/ml collagenase/dispase containing 0.001% DNAse. These lung suspensions were filtered through 40-μm cell strainers, centrifuged, and depleted of red blood cells using RBC lysis buffer. Subsequently, Sca1^+^CD45^−^CD31^−^cells were sorted using MoFlo Astrios (USA).

### Cell culture

Sorted cells were resuspended at a concentration of 1 × 10^6^ cells/ml in DMEM/F12 supplemented with L-glutamine/pyruvate (Invitrogen), 0.5% or 10% fetal bovine serum (FBS) (Gibco, USA), penicillin/streptomycin/amphotericin B, and insulin/transferrin/selenium. Differentiation experiments were carried out using “mesenchymal stem cell functional identification kit” (Cyagen Biosciences Inc., Santa Club, CA, USA) according to the manufacturer’s protocols.

### Quantitative real-time polymerase chain reaction

Total RNA was extracted from cells or homogenized lungs using TRIzol reagent (Invitrogen, CA, USA) according to the manufacturer’s instructions. RNA was reverse-transcribed to generate cDNA using a reverse transcriptase kit (Toyobo, Tokyo, Japan), which was followed by quantitative real-time polymerase chain reaction (RT-PCR) analysis (Toyobo, Tokyo, Japan). The primers were as follows: SP-C: 5′-GGAGCACCGGAAACTCAGAA-3′ (forward), 5′-CTGGCTTATAGGCCGTCAGG-3′ (reverse); club-cell specific protein (CCSP): 5′-ATGAAGATCGCCATCACAATCAC-3′ (forward); 5′-GGATGCCACATAACCAGACTCT-3′ (reverse); FGF10: 5′-TTTGGTGTCTTCGTTCCCTGT-3′ (forward); 5′-TAGCTCCGCACATGCCTTC-3′ (reverse); Ki67: 5′-ATCATTGACCGCTCCTTTAGGT-3′ (forward); 5′-GCTCGCCTTGATGGTTCCT-3′ (reverse); 18s: 5′-CGGCTACCACATCCAAGGAA-3′ (forward); and 5′CCTGTATTGTTATTTTTCGTCACTACCT-3′ (reverse). The relative expression levels of genes were determined by the 2^−ΔΔCT^ method [31] and were normalized to 18s.

### Immunofluorescence staining

Cells were seeded on cell culture chamber slides (NEST, San Diego, CA, USA) and allowed to adhere overnight. They were fixed with 4% paraformaldehyde and then were incubated in 5% BSA and 0.1% Triton X-100 to block nonspecific binding and permeabilize the cell membrane, respectively. Slides were mounted with fluoroshield mounting medium containing DAPI (Abcam, Cambridge, MA, USA) and were viewed on a confocal microscope (FV3000, Olympus, Tokyo, Japan). An anti-mouse pro-SP-C antibody was purchased from Proteintech. An anti-Ki-67-eFluor 570 antibody was obtained from eBioscience (San Diego, CA, USA). An anti-alpha smooth muscle actin (α-SMA) antibody was acquired from Cell Signaling Technology (Danvers, MA, USA), and an anti-CCSP antibody was acquired from Abcam (Cambridge, MA). Anti-aquaporin-5 (AQP5), CD31, and α7nAChR antibodies were obtained from Santa Cruz Biotechnology (Santa Cruz, CA). The secondary antibodies were FITC- and cyanine 3 (Cy3)-conjugated antibodies. All staining procedures were performed with appropriate isotype controls.

### Statistical analysis

Statistical analysis was performed with GraphPad Prism 6 software (GraphPad, San Diego, CA, USA). Student’s *t* tests were utilized unless there were multiple comparisons, in which cases one-way analysis of variance (ANOVA) with Turkey’s correction for post hoc paired comparisons was adopted. All analyses were two sided. The significance level was set at *p* < 0.05. The results are shown as the mean ± standard deviation (SD).

## Results

### LSCs possess multilineage differentiation ability and transdifferentiate into AEC2 under FGF10 stimulation

**Fig. 1:**
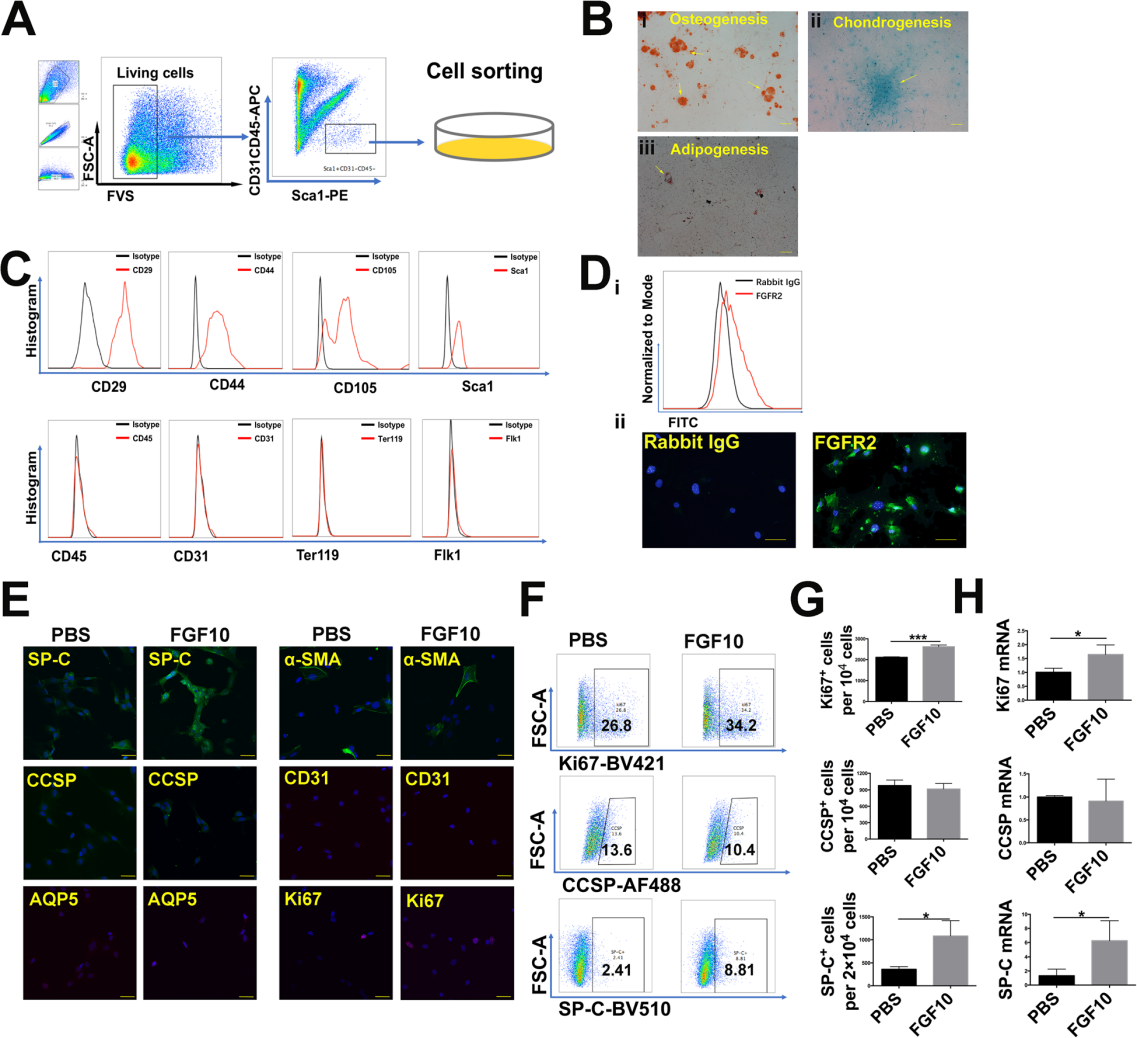
Isolation and characterization of a potential stem cell population from mouse lung. **a** Lung stem cells (LSCs) were sorted by MoFlo Astrios according to the following marker-based sorting strategy: positive for Sca1 and negative for CD45 and CD31. **b** LSCs were induced to differentiate into osteoblasts, chondroblasts, and adipocytes by incubating with defined factors. (i–iii) Differentiation phenotype was confirmed by (i) alizarin red staining of osteoblasts, (ii) alcian blue staining of chondroblasts, and (iii) fat droplets that stained red with oil red; scale bars, 100 μm. **c** Flow cytometry analysis showed that LSCs exhibited characteristics of mesenchymal stem cells: positive for CD29, CD44, CD105, and Sca1 and negative for CD45, CD31, Ter119, and Flk1. **d** Flow cytometry analysis (i) and immunofluorescent staining (ii) results showed that LSCs expressed fibroblast growth factor receptor 2 (FGFR2); scale bars, 200 μm. **e** Immunofluorescent staining results showed that adding 50 ng/mL fibroblast growth factor 10 (FGF10) caused marked induction of SP-C and an increased number of Ki67^+^ cells, but it did not increase AQP5, CCSP, α-SMA, or CD31 levels; scale bars, 40 μm. **f** Flow cytometry analysis showed that FGF 10 caused an increased number of Ki67^+^ cells and SP-C^+^ cells but not CCSP^+^ cells. **g** The statistical analysis of **f**. **h** The relative mRNA expression of Ki67, SP-C, and CCSP was detected by RT-PCR. Data are presented as the mean ± standard deviation (SD). *P < 0.05, ***P < 0.00, assessed by *t* test. AQP5, aquoporin5; CCSP, club-cell specific protein; α-SMA, anti-alpha smooth muscle actin

LSCs (positive for Sca1 and negative for CD45 and CD31) were sorted utilizing the sorting strategy that is shown in Fig. 1a, and then, they were seeded into culture plates. We found that LSCs demonstrated trilineage differentiation abilities: osteogenesis (Fig. 1b (i)), chondrogenesis (Fig. 1b (ii)), and adipogenesis (Fig. 1b (iii)). LSCs also possessed features of mesenchymal stem cells: positive for CD29, CD44, CD105, and Sca1 and negative for CD45, CD31, Ter119, and Flk1 (Fig. 1c). Through limiting dilution analysis, we found that LSCs had self-renewal capacity (Suppl. 1A). These results were consistent with previous investigations [6]. In addition, LSCs expressed FGFR2 (Fig. 1d), the main receptor for FGF10. When treated with FGF10, LSCs elongated and aligned themselves end to end in alveolar-like shapes (Suppl. 1B). We used Ki67 as a cell proliferation marker, since it is present throughout the active phases of the cell cycle (G1, S, G2, and mitosis) but is absent from resting cells (G0) [32]. FGF10 increased the number of AEC2 (labeled by SP-C) and Ki67^+^ cells but not club cells (marked by CCSP) or AEC1 (marked by AQP5, Fig. 1e–h). The results indicated that LSCs maintained self-renewal capacity and possessed multilineage differentiation ability. FGF10 plays a vital role in mediating LSCs proliferation and transdifferentiation.

### Vagus nerve does not influence the decrease in viability and proliferation of LSCs at the early phase (acute inflammation) of LPS-induced lung injury

**Fig. 2:**
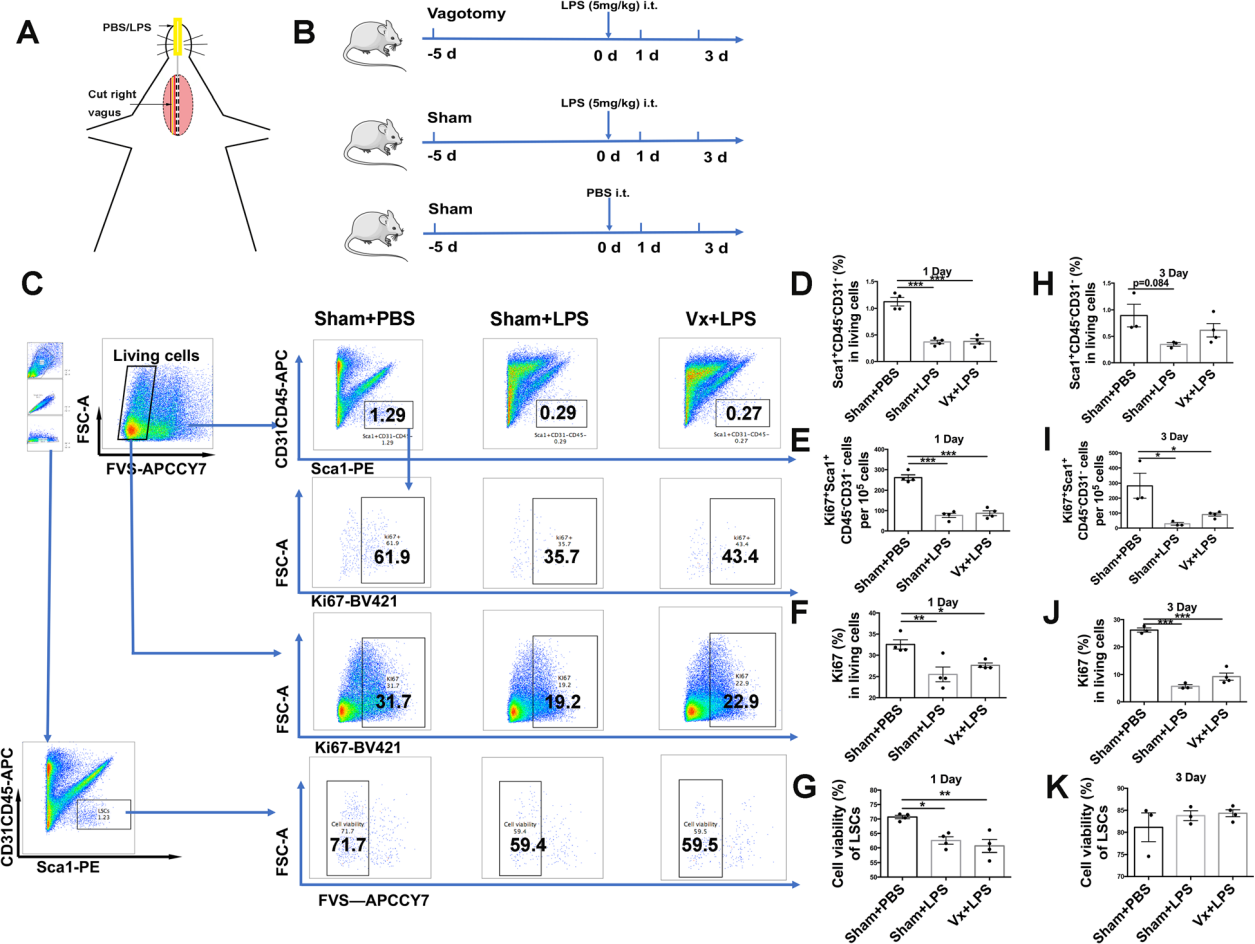
Vagotomy does not influence LSCs expansion at the early phase of LPS-induced lung injury. **a** Schematic model of vagotomy and LPS administration. We cut the right vagal nerves, and then, PBS or LPS was intratracheally delivered. **b** A scheme shows the time of the interventional measures in the model. Mice were vagotomized 5 days before LPS challenge. PBS or LPS (5 mg/kg) was intratracheally delivered to sham or vagotomized mice and was followed up for 1 or 3 days. **c** The gating strategy of LSCs (Sca1^+^CD45^−^CD31^−^), Ki67^+^ LSCs, Ki67^+^ cells, cell viability of LSCs (measured by the fixable viability stain 780 reagent) and their changes in sham or vagotomized mice treated either with PBS or LPS (5 mg/kg) for 1 day. **d**–**g** Changes in LSCs (**d**), Ki67^+^ LSCs (**e**), Ki67^+^ cells (**f**), and cell viability of LSCs (**g**) in sham or vagotomized mice treated either with PBS or LPS (5 mg/kg) for 1 day. **h**–**k** Changes are shown in the numbers of LSCs (**h**), Ki67^+^ LSCs (**i**), and Ki67^+^ cells (**j**), and cell viability of LSCs (**k**) in sham or vagotomized mice treated either with PBS or LPS (5 mg/kg) for 3 days. *N* = 3–4 in each group. Data are presented as the mean ± SD. *P < 0.05, **P < 0.01, and ***P < 0.00, as assessed by one-way ANOVA. i.t., intratracheally; Vx, vagotomy; LPS, lipopolysaccharide

We utilized an LPS-induced lung injury model to investigate the impact of the vagal nerve on LSCs. Mice were vagotomized 5 days before LPS challenge, as shown in Fig. 2a and b. Using the gating strategy shown in Fig. 2c, we found that there were decreased numbers of LSCs (Fig. 2d), Ki67^+^ LSCs (Fig. 2e), and Ki67^+^ cells (Fig. 2f) and a negative impact on viability (Fig. 2g) on the 1st day after LPS (5 mg/kg) challenge. Furthermore, LSCs (Fig. 2h), proliferative LSCs (Fig. 2i), and Ki67^+^ cells (Fig. 2j) were decreased on the 3rd day after LPS (5 mg/kg) challenge, but there was no decrease in cell viability of LSCs (Fig. 2k). These results indicated that LPS could cause loss of LSCs by inhibiting their proliferation at the acute phase of lung injury. However, the worsening effect of a vagotomy was not observed at the early phase of LPS (5 mg/kg)-induced lung injury.

### α7nAChR activation accelerates LSCs expansion and the reparative process at the late phase of LPS-induced lung injury

**Fig. 3:**
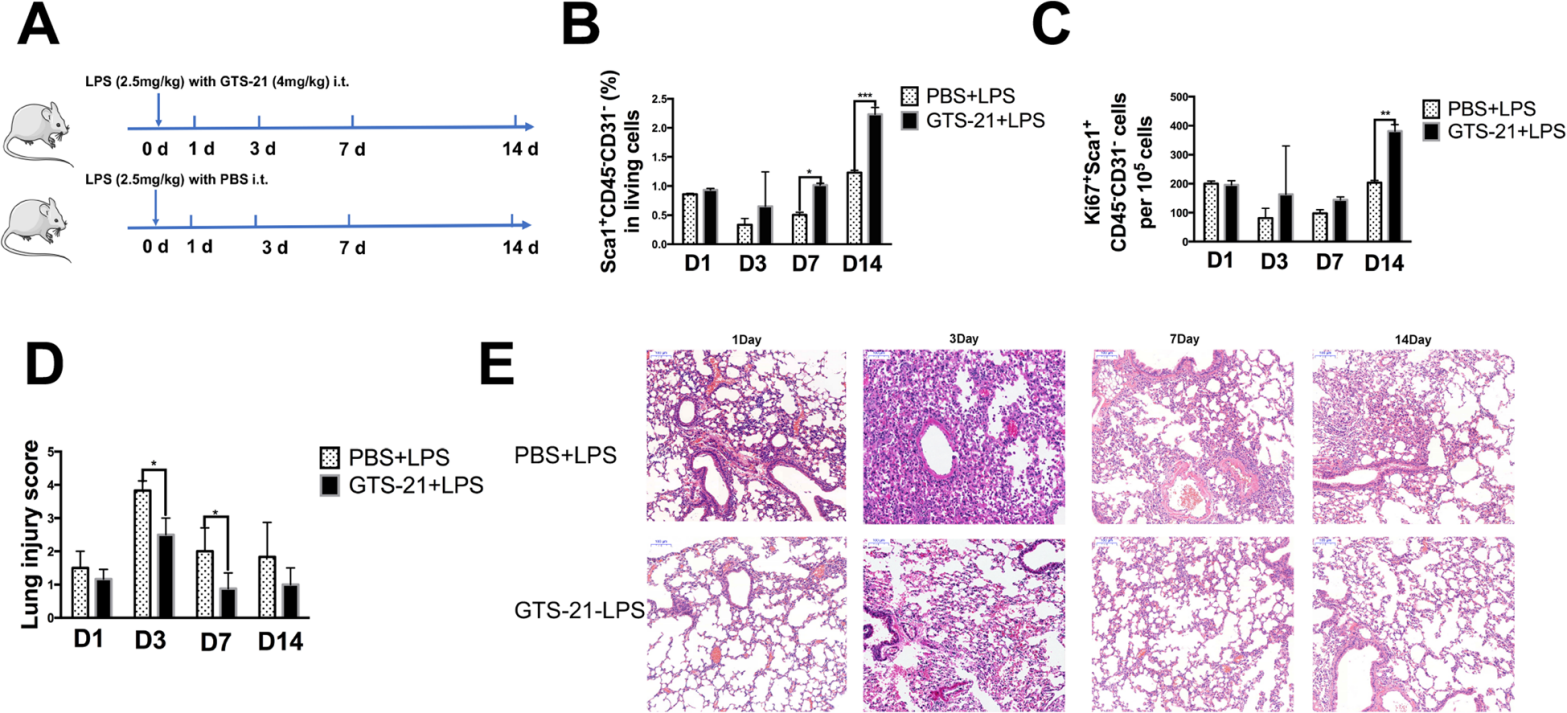
α7nAChR activation accelerates LSCs expansion at the late phase of LPS-induced lung injury. **a** A scheme shows the timing of the interventional measures. LPS (2.5 mg/kg) with PBS or LPS (2.5 mg/kg) with GTS-21 1 (α7nAChR agonist; 4 mg/kg) was intratracheally delivered to mice and was followed up with for 1, 3, 7, or 14 days. **b**–**d** Changes are shown for LSCs (**b**) and Ki67^+^ LSCs (**c**) as well as the lung injury score (**d**) in PBS- or GTS-21 (4 mg/kg)-treated mice after LPS (2.5 mg/kg) challenge at 1, 3, 7, and 14 days. **e** Representative photographs of lung pathologic changes stained by hematoxylin and eosin in PBS- or GTS-21-treated mice after LPS (2.5 mg/kg) challenge at 1, 3, 7, and 14 days; scale bar, 100 μm. *N* = 3–4 in each group. Data are presented as the mean ± SD. *P < 0.05, **P < 0.01, ***P < 0.00, assessed by one-way ANOVA

We proposed that the effect of vagal-α7nAChR signaling on LSCs may be exerted at the late phase of LPS-induced lung injury. Because when using a dose of 5 mg/kg LPS to challenge mice, the Vx + LPS group showed high mortality after the 3rd day, we used a dose of 2.5 mg/kg LPS in the following experiments (Fig. 3a). Importantly, we observed that GTS-21 (α7nAChR agonist) led to the expansion of LSCs and Ki67^+^ LSCs. GTS-21 treatment advanced the recovery time of LSCs from the 14th day to the 7th day after ALI (Fig. 3b) and led to a significantly increased number of Ki67^+^ LSCs on the 14th day and a slightly increased number of Ki67^+^ LSCs on the 7th day (Fig. 3c). Moreover, the degree of pathological injury appeared different in GTS-21-treated mice at later times of ALI, particularly day 3, where there was reduced exudation, decreased epithelial thickness, and a reduction in the number of inflammatory cells (Fig. 3d, e). This implied that α7nAChR activation drove LSCs to proliferate and replenish their population and that they may also transdifferentiate into new alveolar epithelial cells for a long period after lung injury.

### The vagus nerve via α7nAChR promotes LSCs proliferation and transdifferentiation at the late (reparative) phase of LPS-induced lung injury

**Fig. 4:**
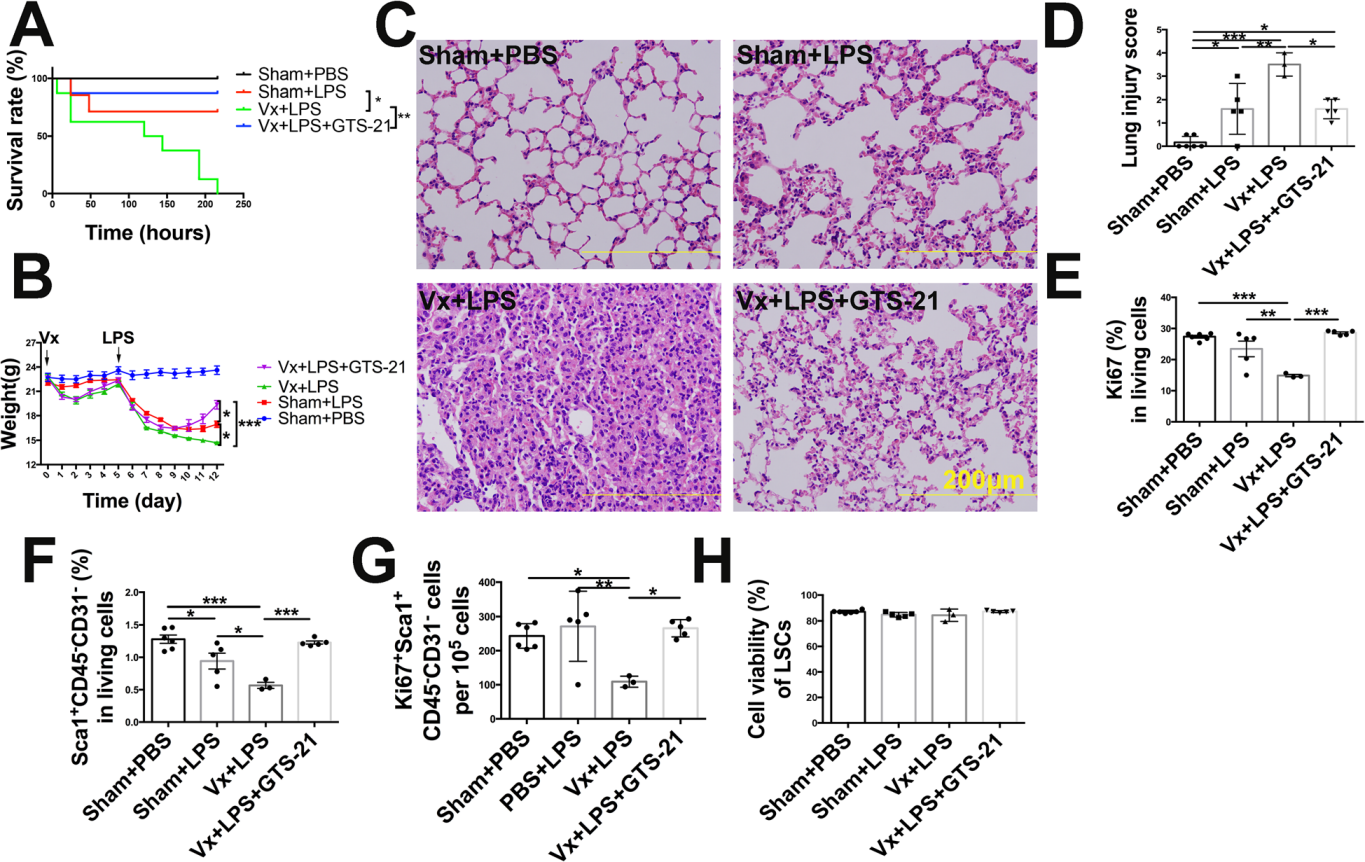
The vagus nerve improves the outcome of lung injury and promotes LSCs proliferation via α7nAChR. **a** Mice received a vagotomy or sham operation 5 days before PBS, LPS (2.5 mg/kg), or LPS+GTS-21 (4 mg/kg) challenge. Kaplan-Meier plot of Sham+PBS, Sham+LPS, Vx+LPS, or Vx+LPS+GTS-21 groups. **b** Changes in mouse body weight in the Sham+PBS, Sham+LPS, Vx+LPS, or Vx+LPS+GTS-21 groups. **c** The lung pathologic injury changes were revealed by hematoxylin and eosin staining in Sham+PBS, Sham+LPS, Vx+LPS, or Vx+LPS+GTS-21 groups; scale bar, 200 μm. **d** The statistical results of the lung injury score. **e** The percentage changes of Ki67^+^ cells gated out of total live lung cells. **f** The percentage changes of Sca1^+^CD45^−^CD31^−^ lung stem cells (LSCs) gated out of total live lung cells. **g** The number of Ki67^+^ LSCs. **h** Cell viability gated for LSCs. *N* = 3–8 in each group. Data are presented as the mean ± SD. *P < 0.05, **P < 0.01, and ***P < 0.00, as assessed by one-way ANOVA

**Fig. 5:**
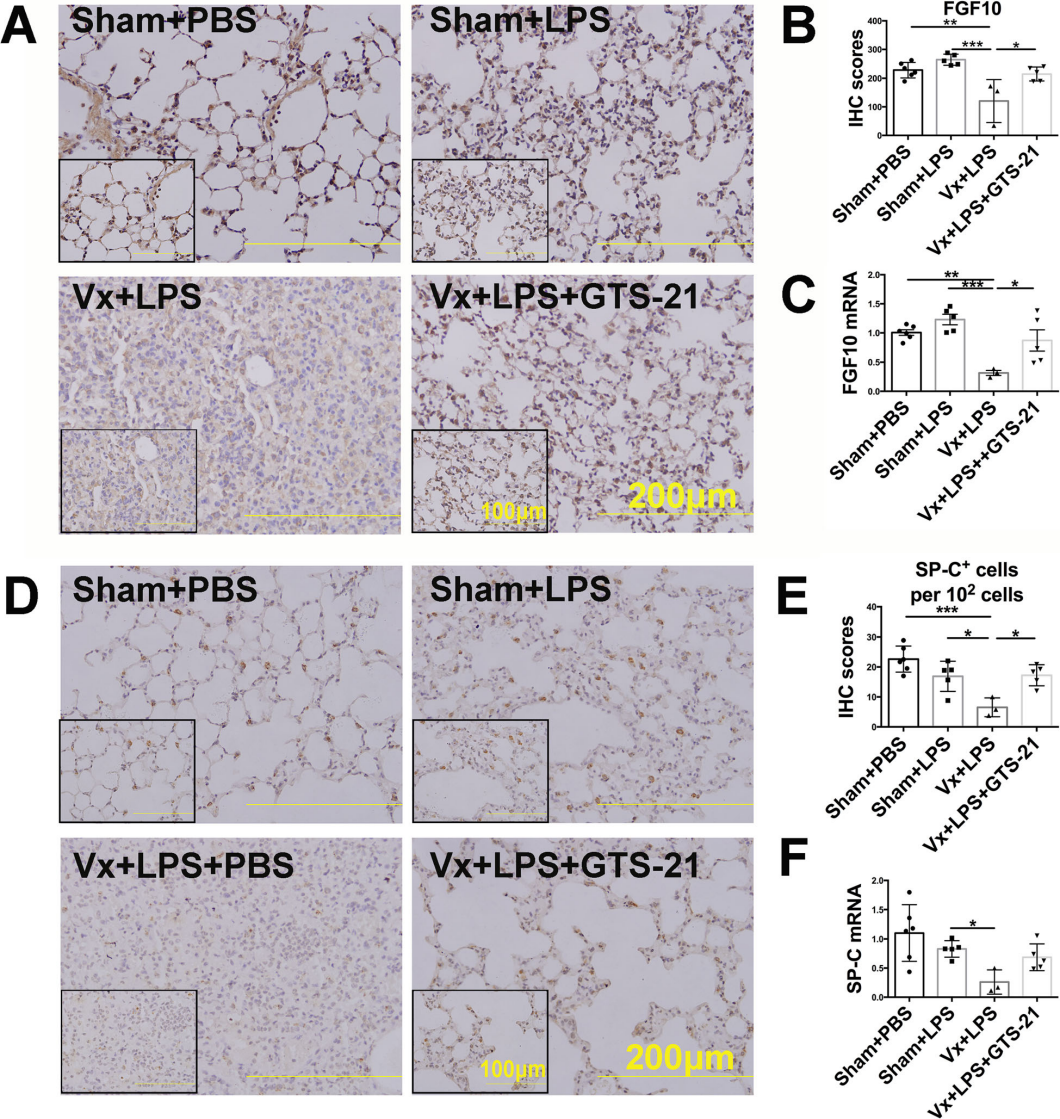
Vagal-α7nAChR signaling upregulates FGF10 expression and enhances LSCs proliferation and transdifferentiation to AEC2. **a** The lung protein expression levels of fibroblast growth factors 10 (FGF10) were measured by immunohistochemistry in the Sham+PBS, Sham+LPS, Vx+LPS, or Vx+LPS+GTS-21 groups. **b** The statistical results of **a**. **c** The relative FGF10 mRNA expression in the lungs of the Sham+PBS, Sham+LPS, Vx+LPS, and Vx+LPS+GTS-21 groups. **d** The SP-C^+^ cells (alveolar epithelial type II cells, AEC2) mass measured by immunohistochemistry in the Sham+PBS, Sham+LPS, Vx+LPS, and Vx+LPS+GTS-21 groups. **e** The statistical results of **d**. **f** The relative SP-C mRNA expression of lung in Sham+PBS, Sham+LPS, Vx+LPS, or Vx+LPS+GTS-21 groups. *N* = 3–6 in each group. Data are presented as the mean ± SD. *P < 0.05, **P < 0.01, and ***P < 0.00, as assessed by one-way ANOVA

We found that vagotomy increased mortality in LPS-challenged mice, which was rescued by administration of GTS-21 (Fig. 4a). In addition, vagotomy significantly decreased body weight by approximately 13.3% on the 12th day, delayed body weight recovery (Fig. 4b), and exacerbated lung pathological damage in LPS-induced lung injury on the 7th day (Fig. 4c, d). GTS-21 reversed vagotomy-induced body weight loss and lung injury (Fig. 4b–d). Moreover, vagotomy led to Ki67^+^ cells and LSCs loss and inhibited Ki67^+^ LSCs without changing the cell viability of LSCs on the 7th day after LPS (2.5 mg/kg) challenge. However, activation of α7nAChR significantly resulted in the expansion of Ki67^+^ cells, LSCs, and Ki67^+^ LSCs (Fig. 4e–h), indicating that the vagus nerve promotes LSCs proliferation and enhances the reparative process via α7nAChR rather than through improving cell survival. Considering that FGF10 was found to promote the proliferation and transdifferentiation of LSCs with in vitro study, we speculated that the protective effect of vagal-α7nAChR signaling was FGF10 dependent. We found that vagotomy decreased the expression of FGF10 protein in the distal lung mesenchyme (Fig. 5a, b) and reduced FGF10 mRNA expression in the total lungs (Fig. 5c), which was accompanied by the loss of SP-C^+^ cells (Fig. 5d, e) and reduction of SP-C mRNA (Fig. 5f) on the 7th day post LPS challenge. Activation of α7nAChR prevented the downregulation of FGF10 and accelerated SP-C^+^ cells repopulation in vagotomized mice at the reparative phase of LPS-induced lung injury (Fig. 5a–f). Collectively, these data supported the idea that the vagus nerve, via α7nAChR, promoted FGF10 expression in the distal lung mesenchyme, which then facilitated LSCs proliferation and transdifferentiation into AEC2. The LSCs expansion induced by α7nAChR activation may be explained by enhanced proliferation prevailing over the transdifferentiation process.

### FGF10 reverses α7nAChR knockout-induced loss of Ki67^+^ LSCs and reduction of AEC2

**Fig. 6:**
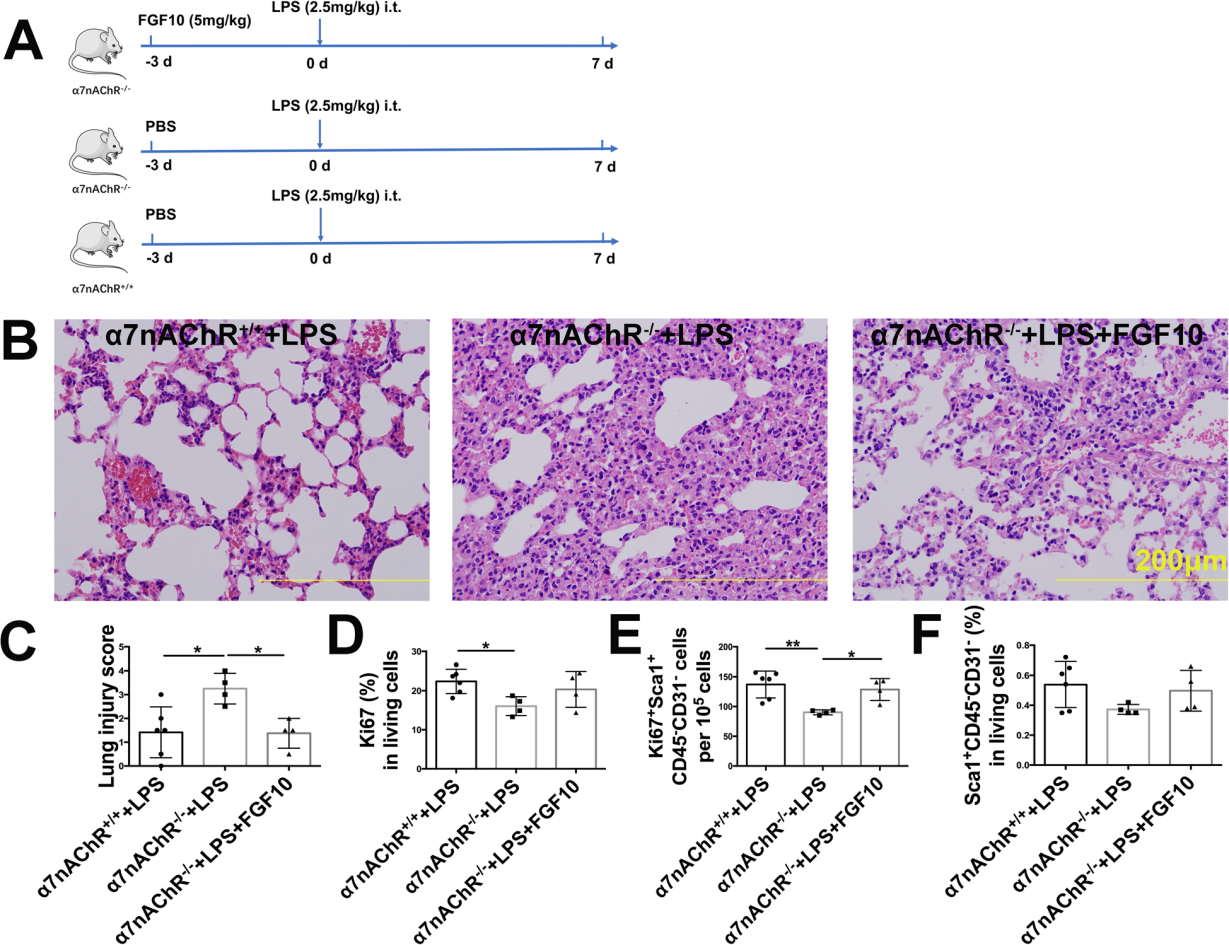
FGF10 reverses α7nAChR knockout-induced loss of Ki67^+^ LSCs. **a** Schematic model of FGF10 administration in LPS-challenged mice. Wild-type (α7nAChR^+/+^) or α7nAChR knockout (α7nAChR^−/−^) mice were intratracheally treated with FGF10 (5 mg/kg) 3 days before LPS (2.5 mg/kg) challenge and then were killed 7 days after LPS challenge. **b** Representative photographs of lung pathologic changes stained by hematoxylin and eosin; scale bar, 200 μm. **c** Changes in the lung injury score. **d** The percentage changes of Ki67^+^ cells gated out of total live lung cells. **e** The number of Ki67^+^ LSCs. **f** The percentage changes of LSCs gated on total lung living cells. *N* = 4–6 in each group. Data are presented as the mean ± SD. *P < 0.05, **P < 0.01, and ***P < 0.00, as assessed by one-way ANOVA

**Fig. 7:**
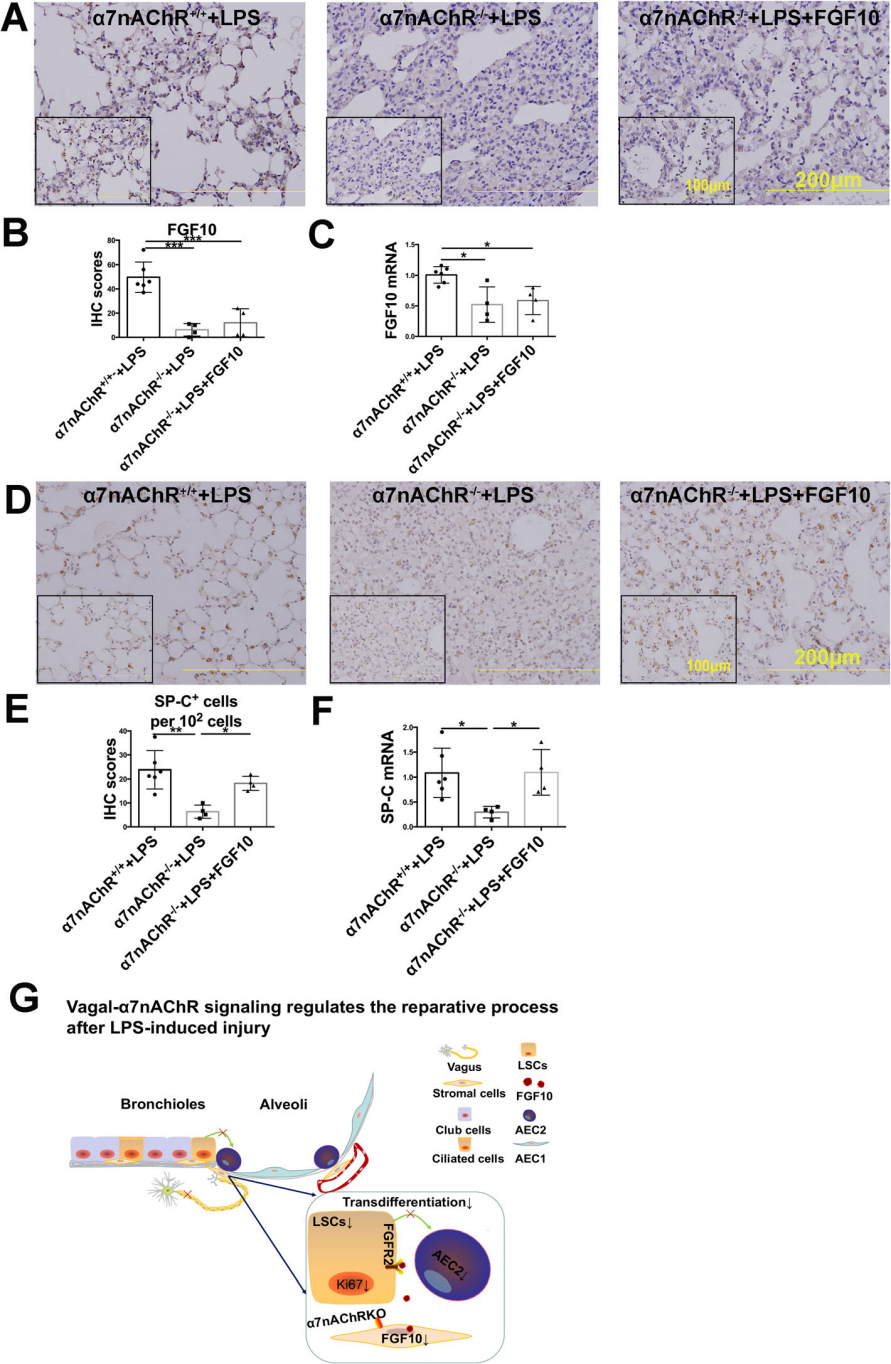
FGF10 is essential for α7nAChR-induced LSCs transdifferentiation to AEC2. **a** Wild-type (α7nAChR^+/+^) or α7nAChR knockout (α7nAChR^−/−^) mice were intratracheally treated with FGF10 (5 mg/kg) 3 days before LPS (2.5 mg/kg) challenge and then were killed 7 days after LPS challenge. Lung FGF10 expression was examined by immunohistochemistry staining. **b** Changes in the FGF10 immunohistochemistry score. **c** Relative gene expression of FGF10. **d** Immunohistochemistry evaluation of SP-C^+^ cells in the lung. **e** The statistical results of the evaluation of SP-C^+^ cells. **f** The relative gene expression of SP-C. **g** Schematic model of the vagal α7nAChR-mediated regenerative process after LPS-induced lung injury. *N* = 4–6 in each group. Data are presented as the mean ± SD. *P < 0.05, **P < 0.01, and ***P < 0.00, as assessed by one-way ANOVA

The aforementioned findings indicate that activation of α7nAChR induces LSCs proliferation and transdifferentiation with activation of the FGF10 pathway engaging with vagal signals. To further confirm whether FGF10 plays an important role in this process, we used α7nAChR knockout mice and designed an FGF10 rescue assay (Fig. 6a). We found that FGF10 administration partially reversed the α7nAChR deletion-induced deteriorated lung damage (Fig. 6b, c). Furthermore, α7nAChR ablation resulted in a significant loss of Ki67^+^ cells (Fig. 6d) and Ki67^+^ LSCs (Fig. 6e) and a downward trend of the number of LSCs (Fig. 6f) over 7-day period after LPS challenge. Importantly, α7nAChR deletion-induced Ki67^+^ LSCs loss was significantly rescued by FGF10. FGF10 protein and mRNA levels were decreased in α7nAChR knockout mice (Fig. 7a–c), suggesting that activation of α7nAChR promotes LSCs proliferation and transdifferentiation via the FGF10 pathway. In addition, LPS-challenged α7nAChR knockout lungs exhibited a decreased number of AEC2 labeled by SP-C (Fig. 7d–f). FGF10 reversed α7nAChR knockout-induced loss of AEC2. These data supported the concept that LSCs deploy α7nAChR-FGF10 signaling to control their fate including proliferation and transdifferentiation.

## Discussion

The adult lung is a quiescent tissue under normal physiologic conditions, but it responds efficiently to injury by activating the proliferation and transdifferentiation of stem/progenitor cells to reconstitute the alveolar epithelium and promote recovery [33]. However, whether this regenerative process leads to normal or dysplastic repair is dependent on several factors, and we herein provide additional insights into this process. We clarified that the vagus nerve via α7nAChR leads to LSCs proliferation and transdifferentiation and accelerates the reparative process after lung injury. LSCs proliferation and transdifferentiation during the reparative process of LPS-induced lung injury consists of transmission of the vagus nerve, lung α7nAChR activation, and FGF10-dependent improved proliferation and transdifferentiation in LSCs. Moreover, vagal-α7nAChR signaling was proven to be an activator of the FGF10 pathway. Thus, based on the present findings, we conclude that vagal-α7nAChR signaling regulates LSCs in an FGF10-dependent manner, which leads to lung stem cells proliferation and transdifferentiation after lung injury.

Depending on the type and severity of lung injury, different cell types are involved in the reparative process after lung injury [33–35]. It has been reported in previous investigations that vagal-α7nAChR signaling protects against various acute lung injuries [13, 17, 18]. To address whether this signaling affects stem cell-mediated lung repair, we comprehensively examined the changes in lung stem cell markers in LPS-challenged vagotomized mice or α7nAChR-knockout mice. We found that the number of lung stem cells in Sca1^+^CD45^−^CD31^−^ populations of cells was reduced after LPS-induced lung injury, and an obvious expansion of potentially regenerative Sca1^+^CD45^−^CD31^−^ cells was observed in the mouse lung 1 to 2 weeks after LPS challenge. In addition, administration of an α7nAChR agonist (GTS-21) accelerated the proliferation and expansion of Sca1^+^CD45^−^CD31^−^ cells during the reparative process after LPS-induced lung injury.

Sca1^+^CD45^−^CD31^−^ cells were originally identified as BASCs [1]. However, some studies demonstrated that Sca1^+^CD45^−^CD31^−^ cells comprise a heterogeneous population of progenitors, which are mainly lung mesenchymal progenitors [5, 10] or a population of multipotent stem cells [6]. The divergence concerning the phenotype of Sca1^+^CD45^−^CD31^−^ cells was explained by different antibodies obtained from different companies that showed variability in the cell populations isolated according to these markers. We similarly assessed this cell population after isolation. Consistent with Hegab’ study [6], we observed that LSCs possessed self-renewal capacity and possessed the characteristics of mesenchymal stem cells and lung epithelial stem cells. Hence, Sca1^+^CD45^−^CD31^−^ cells are bona fide lung stem cells and can be used to assess the lung regeneration and reparative processes. Importantly, we found that vagotomy increased mortality, delayed body weight recovery, and exacerbated lung pathological damage in LPS-induced lung injury mouse models. The application of an α7nAChR agonist reversed the vagotomy-induced high mortality, body weight loss, and lung injury. Furthermore, vagotomy led to LSCs loss by inhibiting their proliferation and reduced the number of SP-C^+^ cells, but this effect could be rescued by GTS-21 administration. These data support the idea that vagal-α7nAChR signaling regulates the proliferation of LSCs and promotes the regenerative process after injury.

Several lines of evidence have highlighted the importance of FGF10 in lung regeneration regulation. FGF10 overexpression has been reported to enable basal stem cells to repopulate the noncartilaginous airways [36]. Moreover, FGF10-FGFR2B signaling promoted the expansion of basal cells and alveolar epithelial regeneration after lung injury [37]. In this study, we observed that vagotomy decreased the expression of FGF10 in the distal lung mesenchyme, which was accompanied by the loss of SP-C^+^ cells at the reparative phase after LPS challenge. Activation of α7nAChR could prevent the downregulation of FGF10 and the loss of SP-C^+^ cells. Supplementation with FGF10 could prevent α7nAChR knockout-induced lung injury and α7nAChR knockout-induced the loss of Ki67^+^LSCs and SP-C^+^ cells during LPS-induced lung injury. Previously, we also demonstrated that α7nAChR deficiency could increase proinflammatory cytokines in lung injury caused by LPS challenge [17, 18] and that FGF-10 could mobilize mesenchymal stem cells and reduce lung inflammatory cytokines [38]. In addition, it has been reported that FGF10 is also expressed on a population of lipofibroblasts that are spatially associated with AEC2 cells [39]. These findings are consistent with FGF10 immunofluorescent staining in this study. Collectively, these data indicate that vagal-α7nAChR signaling enhances FGF10 expression in the lung mesenchyme and that FGF10 in turn promotes LSCs proliferation and transdifferentiation to AEC2. In addition, we also confirmed that FGF10 promotes LSCs proliferation and transdifferentiation in vitro in our study. Meanwhile, vagus nerve-induced proliferation prevails over transdifferentiation during the expansion of LSCs.

Retardation of regeneration in both the liver [40] and pancreatic islet [24] and impairment of extremity regeneration [41, 42] due to surgical denervation have been widely observed. Support for the idea that tissue regeneration is governed by neuronal signaling has been demonstrated for a few decades. However, the exact mechanisms remain unknown. This is the first study that clarifies the positive effect of vagal-α7nAChR signaling at the reparative phase after lung injury. We found that the vagus nerve, via α7nAChR, promotes lung stem cell proliferation and expansion in an FGF10-dependent manner. Thus, vagus stimulation and α7nAChR agonist might be potent therapeutic targets for treating lung injury. The concept of vagal-α7nAChR signaling governing lung stem cell proliferation may open a new avenue of research in the field of lung regeneration.

## Conclusion

Vagal-α7nAChR signaling promotes lung stem cell (Sca1^+^CD45^−^CD31^−^) expansion and accelerates the reparative process of lung injury, and this process is FGF10 dependent. Vagus nerve α7nAChR-mediated lung injury repair holds potential for the development of novel approaches to lung stem cell therapy. A schematic image showing that vagal-α7nAChR signaling regulates the reparative process after lung injury is showed in Fig. 7g.

## Supplementary information

**Additional file 1: Figure S1.** LSCs possess self-renewal ability. A. In the LDA assay, we observed that 25.61% ± 5.79% of the wells contained colonies. In addition, seeding of single cells that originated from secondary clones could generate tertiary clones, further confirming their self-renewal ability. Daughter colonies were always morphologically identical to primary cell colonies. Clones could be cryopreserved. And when re-cultured, their growth kinetics or morphology were not changed. B. When treated with FGF10, LSCs elongated and aligned themselves end to end in alveolar-like shapes within 2–3 days.

## Data Availability

All data generated or analyzed during this study are included in this published article.

## Abbreviations

LSCs: Lung stem cells
Sca1: Stem cell antigen 1
α7nAChR: Alpha7 nicotinic acetylcholine receptor
LPS: Lipopolysaccharide
FGF10: Fibroblast growth factor 10
RT-PCR: Real-time polymerase chain reaction
AEC1: Alveolar epithelial type I cells
AEC2: Alveolar epithelial type II cells
ARDS: Acute respiratory distress syndrome
FGFs: Fibroblast growth factors
FGFR2: Fibroblast growth factor receptor 2
PE: Phycoerythrin
Cy3: Cyanine 3 (Cy3)
FVS780: Fixable viability stain 780
APC: Allophycocyanin
FITC: Fluorescein isothiocyanate
Vx: Vagotomy
PBS: Phosphate buffer saline
SP-C: Surfactant protein C
CCSP: Club-cell specific protein
AQP5: Aquoporin5
α-SMA: Alpha smooth muscle actin
SD: Standard deviation
