# Supplementary material for: Vagal-α7nAChR signaling promotes lung stem cells regeneration via fibroblast growth factor 10 during lung injury repair
Source: Stem Cell Res Ther. 2020 Jun 10;11:230. doi: 10.1186/s13287-020-01757-w (PMC7288553; doi:10.1186/s13287-020-01757-w)
Supplement: Supplementary file 1 — Additional file 1: Figure S1. LSCs possess self-renewal ability. A. In the LDA assay, we observed that 25.61% ± 5.79% of the wells contained colonies. In addition, seeding of single cells that originated from secondary clones could generate tertiary clones, further confirming their self-renewal ability. Daughter colonies were always morphologically identical to primary cell colonies. Clones could be cryopreserved. And when re-cultured, their growth kinetics or morphology were not changed. B. When treated with FGF10, LSCs elongated and aligned themselves end to end in alveolar-like shapes within 2–3 days. [file 13287_2020_1757_MOESM1_ESM.docx]

**Supplemental Figure** 1

**Supplemental methods**

**Limiting dilution analysis (LDA)**

LDA was performed as descried(1). Firstly, we diluted LSCs to 1 cell per 100 μL in 96-well plates. Then, wells that contained single cells were marked and examined daily to observe we observed colony-forming ability under microscope (empty wells or wells that contained >1 cell were ignored). This was repeated 3 times. Picked up some colonies that originated from single cells, then dissociated and re-cultured in another 96-well plate to examine their ability to produce secondary and tertiary colonies.

**Differentiation of LSCs in vitro**

Sorted cells were resuspended at a concentration of 1 ×10^6^ cells/ml in DMEM/F12 supplemented with L-glutamine/pyruvate (Invitrogen), 10% FBS, penicillin/streptomycin/amphotericin B, and insulin/transferrin/selenium. LSCs were treated with 50 ng/mL of fibroblast growth factor 10 (FGF10) for 2-3 days.

1. Hegab, A. E., Kubo, H., Fujino, N., Suzuki, T., He, M., Kato, H., and Yamaya, M. (2010) Isolation and characterization of murine multipotent lung stem cells. *Stem Cells Dev* **19**, 523-536

**Results:**

A B


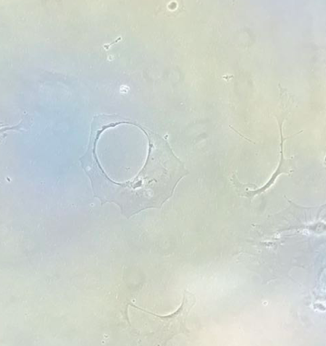


**FigureS1 LSCs possess self-renewal ability**. A. In the LDA assay, we observed that 25.61% ± 5.79% of the wells contained colonies. In addition, seeding of single cells that originated from secondary clones could generate tertiary clones, further confirming their self-renewal ability. Daughter colonies were always morphologically identical to primary cell colonies. Clones could be cryopreserved. And when being re-cultured, their growth kinetics or morphological were not changed. B. When treated with FGF10, LSCs elongated and aligned themselves end to end in alveolar-like shapes within 2-3 days
